# Distinct Peripheral Blood RNA Responses to *Salmonella* in Pigs Differing in *Salmonella* Shedding Levels: Intersection of IFNG, TLR and miRNA Pathways

**DOI:** 10.1371/journal.pone.0028768

**Published:** 2011-12-12

**Authors:** Ting-Hua Huang, Jolita J. Uthe, Shawn M. D. Bearson, Cumhur Yusuf Demirkale, Dan Nettleton, Susan Knetter, Curtis Christian, Amanda E. Ramer-Tait, Michael J. Wannemuehler, Christopher K. Tuggle

**Affiliations:** 1 Department of Animal Science, Iowa State University, Ames, Iowa, United States of America; 2 National Animal Disease Center, United States Department of Agriculture- Agricultural Research Service, Ames, Iowa, United States of America; 3 Department of Statistics, Iowa State University, Ames, Iowa, United States of America; 4 College of Veterinary Medicine, Iowa State University, Ames, Iowa, United States of America; Virginia Commonwealth University, United States of America

## Abstract

Transcriptomic analysis of the response to bacterial pathogens has been reported for several species, yet few studies have investigated the transcriptional differences in whole blood in subjects that differ in their disease response phenotypes. *Salmonella* species infect many vertebrate species, and pigs colonized with *Salmonella enterica* serovar Typhimurium (ST) are usually asymptomatic, making detection of these *Salmonella*-carrier pigs difficult. The variable fecal shedding of *Salmonella* is an important cause of foodborne illness and zoonotic disease. To investigate gene pathways and biomarkers associated with the variance in *Salmonella* shedding following experimental inoculation, we initiated the first analysis of the whole blood transcriptional response induced by *Salmonella*. A population of pigs (n = 40) was inoculated with ST and peripheral blood and fecal *Salmonella* counts were collected between 2 and 20 days post-inoculation (dpi). Two groups of pigs with either low shedding (LS) or persistent shedding (PS) phenotypes were identified. Global transcriptional changes in response to ST inoculation were identified by Affymetrix Genechip® analysis of peripheral blood RNA at day 0 and 2 dpi. ST inoculation triggered substantial gene expression changes in the pigs and there was differential expression of many genes between LS and PS pigs. Analysis of the differential profiles of gene expression within and between PS and LS phenotypic classes identified distinct regulatory pathways mediated by IFN-γ, TNF, NF-κB, or one of several miRNAs. We confirmed the activation of two regulatory factors, SPI1 and CEBPB, and demonstrated that expression of miR-155 was decreased specifically in the PS animals. These data provide insight into specific pathways associated with extremes in *Salmonella* fecal shedding that can be targeted for further exploration on why some animals develop a carrier state. This knowledge can also be used to develop rational manipulations of genetics, pharmaceuticals, nutrition or husbandry methods to decrease *Salmonella* colonization, shedding and spread.

## Introduction

*Salmonella enterica* serovar Typhimurium (ST) infects almost all vertebrates, including reptiles, birds, and mammals [1]. In humans, ST causes an acute gastroenteritis known as salmonellosis. *Salmonella* colonization of pigs can lead to an enterocolitis of variable severity with the bacteria often establishing a carrier status in the host [2]. The decreased performance of pigs with subclinical *Salmonella* infections has a negative economic impaction the swine industry [3]. Moreover, pigs that persistently shed *Salmonella* pose a significant threat to public health by increasing the potential for foodborne disease [2], [4], [5]. To reduce the incidence and severity of salmonellosis and other infectious diseases, a need exists to define the immune genes and pathways responsible for enhanced disease resistance and pathogen clearance [6]. Genetic selection for improved humoral and cell-mediated immunity to develop pigs with enhanced disease resistance has been reported [7], while heritabilities of specific immune component parameters have been estimated and correlations to performance traits defined [8], [9], [10], [11]. Genomic regions controlling leukocyte numbers and response to mitogens have also been identified [12], [13], [14]. In a *Salmonella* challenge experiment, van Diemenet al. found evidence for genetic control of innate immunological traits (e.g., numbers and function of polymorphonuclear leukocytes) and associated some of these with susceptibility to salmonellosis [15], [16].

An alternative approach to this problem is to identify the genes that respond to *Salmonella* at the RNA level, and that are correlated with decreased fecal shedding of *Salmonella*. Such genes would then provide valuable selectable biomarkers for decreased disease spread and potentially for improved innate immune responsiveness to bacterial pathogens [17]. Initial studies have focused on measuring host response to *Salmonella* spp. [18], [19], [20], [21], [22]. Screening for novel host mRNA responses to *Salmonella* has also been reported [23]. More global analyses of the response to *Salmonella* in immune tissues such as lung, Peyer's patch, or lymph node using Q-PCR assays [24] or microarrays [25], [26] have also been published. However, such analyses are difficult to translate into biomarker development because tissues are collected at slaughter. Optimally, this type of analysis needs to be performed on samples that are easy and inexpensive to collect from many live animals. One such sample would be whole blood, and measuring the transcriptome of whole blood to survey human immune responses to various diseases has become an accepted method to identify biomarkers associated with disease [27]. The transcriptomic response of peripheral blood mononuclear cells (PBMC) to bacteria, virus and immune stimulants has been investigated; effective classifiers were built to distinguish the infected or non-infected status of the patient, as well as etiology of the infection [28], [29], [30]. These studies indicate that measuring the blood transcriptome may be useful in identifying genes controlling the variability in disease resistance in the pig.

We hypothesize that there are yet unidentified host genetic differences controlling phenotypic variation of *Salmonella* shedding (and thus transmission) in pigs. Such postulated genetic differences may control the effectiveness of early innate immune responses, and we predict that these differences are likely to be most distinct in individuals at the extreme ends of *Salmonella* shedding. These differences may be reflected in the variation of gene expression response to *Salmonella* inoculation among animals with distinct ST fecal shedding counts. However, no data exist on the whole blood transcriptomic response to *Salmonella*. Therefore, we have initiated research to determine the variation in transcriptional responses to ST across 40 pigs [31]. An initial characterization of these animals found a significant positive correlation between serum interferon-γ (IFN-γ), levels at 2day post-inoculation (dpi) and ST fecal shedding levels at two and seven dpi. In the current study, *Salmonella* fecal shedding data and whole blood transcriptome profiling of a subset of this pig population were employed to 1) define extreme shedding phenotype classes of pigs based on their total number of *Salmonella* shed during the experiment; 2) determine the global gene expression responses in porcine whole blood in response to ST colonization; and 3) identify the gene expression differences subsequent to ST inoculation between two defined groups, low shedding (LS) and persistently shedding (PS) pigs. This study describes for the first time the whole blood transcriptomic response to ST inoculation in pigs and provides regulatory pathway information on the differences between animals that shed large numbers of *Salmonella* following inoculation as compared to animals that shed much less *Salmonella*.

## Materials and Methods

### Sample collection, fecal bacteria quantification and selection of animals

Peripheral blood samples were collected from two pig populations which were challenged similarly with ST: challenge population #1 of 40 pigs which has been previously described [31], and challenge population #2 of 77 pigs which is described here. In brief, for both populations, the piglets used were from sows (crossbred or Yorkshire breeds) and bred to boars from several different breeds. All available breed information is shown in Supplemental Table S1. Piglets were raised in climate-controlled, fully enclosed isolation facilities at the USDA-ARS-National Animal Disease Center (NADC) in Ames, IA. The pigs tested fecal negative for *Salmonella* three times before intranasal challenge with 10^9^ colony forming units (cfu) of nalidixic acid resistant ST χ4232 at 7 weeks of age. Fecal and blood samples were collected from each animal at 0, 2, 7, 14, and 20 dpi. *Salmonella* was quantified from feces by direct counting using bacteriological methods as described by Uthe et al. [31]. Peripheral whole blood (approximately 2.3 mL) was collected from the jugular vein into PAXgene Blood RNA tubes and processed according to the instructions of QIAGEN. All procedures involving animals were lawful and approved by the USDA-ARS-NADC Animal Care and Use Committee (approval ID: ACUP #3586).

Pigs were selected for RNA analysis from the phenotypic extremes of the *Salmonella* shedding data initially from challenge population #1 as follows: the shedding class phenotype was defined based on the total fecal excretion of ST as determined by calculating cumulative area under the plotted log curve (AULC) of logarithmically normalized fecal counts obtained between day 0 to day 20 post-inoculation for each animal. Based on the AULC, pigs of extreme shedding phenotype were identified as low shedders (LS1) and persistent shedders (PS1) and were selected from challenge population #1 for microarray analysis. Where possible, littermates were chosen that exhibited different shedding phenotype. Additional sets of extreme animals were selected using the same criteria from the remaining animals in the challenge population #1 (LS2 and PS2), and also from challenge population #2 (LS3 and PS3; LS4 and PS4).

### RNA preparation of selected animals

Total RNA was prepared from 4.5–9.0 ml of solution from the PAXgene Blood RNA tubes for LS1 and PS1pigs at day 0, 2 and 20. Samples at day 0 and day 2 were collected for LS2, PS2, LS4, and PS4 animals (50–100% of the total volume) using the PAXgene Blood RNA kit (Qiagen, Cat. no. 762164). The DNA was removed by in-solution DNase I digestion and RNeasy mini elute kit cleanup as recommended by QIAGEN. PCR assay without reverse transcription was used to confirm that the RNA samples were DNA-free. The quantity and quality of the RNA were determined using Agilent 2100 Bioanalyzer (Agilent Technologies, Santa Clara, CA) and Nanodrop 2000 (Thermo Scientific, Wilmington, DE). RNA samples with RIN number lower than 7 or yield less than 3 µg was identified as low quality and excluded from the experiment.

For microRNA analysis, total RNA was isolated from PAXgene tubes for LS3 and PS3 animals using PAXgene Blood miRNA kit (Qiagen, Cat. no. 763134), and the miRNA quality was confirmed using Agilent 2100 Bioanalyzer.

### Microarray hybridization and statistical analysis

The porcine genome microarrays were purchased from Affymetrix (Cat. no. 900623; Santa Clara, CA). The RNA labeling, porcine gene chip hybridization, washing and signal detection were done at the GeneChip Facility, Iowa State University, Ames IA according to the manufacturer's instructions. The Bioconductor package *affy* in R was used to compute normalized MAS5.0 expression measures from the 20 Affymetrix GeneChips. A linear mixed model including fixed effects for shedding status, time and interaction between shedding status and time along with random pig effects was fit to the expression data for each gene using SAS PROC MIXED. As part of each linear model analysis, p-values were obtained for the shedding status-by-time interaction test, tests for changes over time within and averaged over low and persistent shedding groups and tests for a shedding effect at each time point and averaged over time points. The p-values for each test were converted to q-values for false discovery rate estimation [32]. All Affymetrix data has been submitted to GEO (GSE27000).

### Globin RNA removal testing

Samples from two pigs were used to test a need for the removal of globin RNA in Affymetrix microarray hybridizations. Globin RNA from two test samples was removed using the globin reduction protocol adapted from Affymetrix (www.affymetrix.com). Subsequently, total RNA was cleaned-up using GeneChip sample cleanup module (Affymetrix). Efficiency of globin RNA removal was confirmed by real-time RT-PCR to amplify porcine alpha and beta globin cDNAs using the QuantiTect SYBR Green RT-PCR Kit (Qiagen, Valencia, CA) and the Chromo4 Real-Time PCR Detection System (BioRad Laboratories, Hercules, CA). To ensure that the globin RNA removal procedure did not affect the expression levels of other transcripts, cDNAs coding for HSPH1, DNAJA4, G3PDH, RPL32 and CXCL10, were amplified before and after globin RNA removal by real-time RT-PCR using primers reported in by Uthe et al. [17]. Expression levels of these genes were not affected by globin RNA removal (J.U., S.M.D.B and C.K.T., data not shown). Analysis of the effect of globin removal on Affymetrix microarray hybridization data was also performed and included number of present, marginal and absent calls; number of genes with detection *p*-value<0.05 and an estimate of whole chip signal intensities. No dramatic changes were observed (T.H., J.U., and C.K.T., data not shown). While previous reports have indicated that removal of globin RNA can improve transcriptome profiling of whole blood RNA [33], [34], others have not used globin reduction methods [35]. As our results did not indicate an obvious benefit of globin RNA removal prior to Affymetrix microarray analysis at either the chip level or at an individual gene level, globin RNA was not removed prior to hybridization.

### Real-time PCR

Blood RNA samples from the 10 animals, LS1 and PS1, which have been used for the Affymetrix experiment, plus RNA samples from a second set of 10 animals (LS2 and PS2) were analyzed by real-time PCR. Two additional sets of animals (LS3 and PS3; LS4 and PS4) from challenge population #2 also have been analyzed by real-time PCR. The reverse transcription was performed using SuperScript II Reverse Transcriptase and Oligo(dT) primer according to the manufacturer's instructions (Invitrogen, Carlsbad, CA). All reverse transcription reactions were run along with “no-template controls”. The no-template controls gave non-detectable signals in all samples, confirming the high specificity of the assays. Real-time PCR was performed using a standard SYBR Green PCR kit (Applied Biosystems, Carsbad, CA) and BIO-RAD iQ5 Real-Time PCR Detection System. All reactions were run in duplicate. The data were normalized initially using the gene YWHAZ (tyrosine 3-monooxygenase/tryptophan 5-monooxygenase activation protein, zeta polypeptide) or RPL32 (ribosomal protein L32). However, normalization with either of those two RNA levels or the average of these RNA levels did not correct any systematic bias between samples (as measured by reduced variance). In fact, higher variance was introduced compared to normalization to input nucleic acid (data not shown) Thus, results were normalized only to the total input RNA or cDNA used, as we have reported previously [36]. The significance level was set to 0.05.

The expression level of miR-124 and -155 were quantified in the LS3 and PS3 animals using the Stem-loop TaqMan MicroRNA Assay (ABI Assay: 000446 and 002623) and normalized by the total amount of input RNA. MiRNA quantification assays were performed only with the LS3 and PS3 animals because the blood samples for challenge population #1 have been completely used for standard RNA preparation.

### Microarray probeset annotation and differentially expressed gene functional annotation

The most current porcine Affymetrix Genechip annotation was used to assign probesets to RefSeq ID [37]. Differentially expressed genes (q value<0.05 or 0.1, fold change>1.5 or <0.66) were divided into categories of different expression pattern as a result of ST inoculation. The open-access bioinformatics tool InnateDB [38] was used to identify significantly regulated pathways between different time points and shedding phenotypes. The web-based program DAVID [39] was used to analyze the function of differentially expressed gene identified by microarray. The significantly enriched gene expression regulators of these differentially expressed gene lists were identified by Sub-Network Enrichment Analysis (SNEA) using Pathway Studio®.

## Results

### Classification of pigs as low shedders or persistent shedders of *Salmonella* after challenge

**Figure 1:**
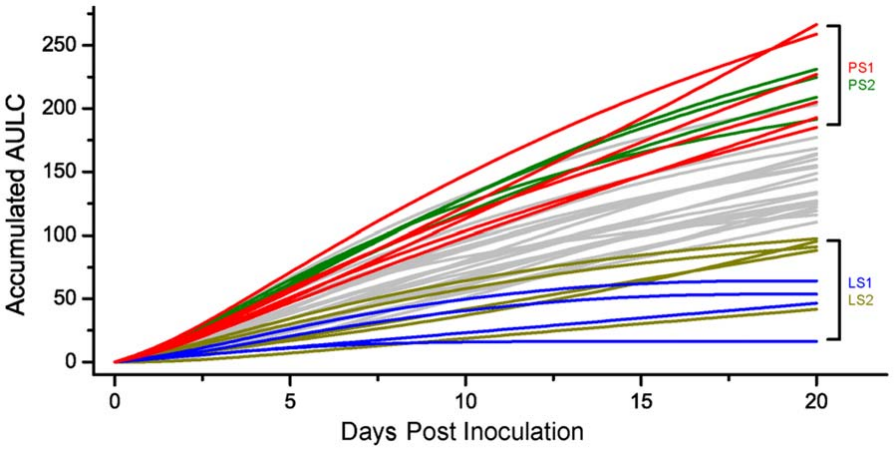
Area under the log curve (AULC) defines extremes for *Salmonella* shedding phenotypes. The line graph illustrates the AULC accumulated over time for each animal. At each time point, the AULC accumulated up to that time point for each animal was plotted; i.e., the values shown at day 7 represent the AULC from day 0 to day 7; the total AULC shown at day 20 (AULC accumulated from day 0 to day 20) is the phenotype used for shedding class distinction. Groups of pigs representing the two extremes for AULC at day 20 dpi, were thus selected for microarray analysis (LS1 and PS1) and qPCR (LS2 and PS2). Animals in the LS1 group are in blue; PS1 animals are in red, while LS2 animals and PS2 animals are shown in yellow green and green, respectively. Data for the non-selected animals are shown in gray.

In challenge population #1, 40*Salmonella* fecal-negative crossbred pigs were inoculated with *Salmonella enterica* serovar Typhimurium [31]. As described by Uthe and colleagues, bacteriological evaluation of fecal samples indicated all 40 pigs were shedders of *Salmonella* at 2 dpi, with an increase in body temperature (fever). A wide range of *Salmonella* shedding counts was observed during the 20-day experiment, ranging from quantitatively undetectable to 320,000 cfu/g feces. To develop a new phenotypic measure for categorizing pigs at the extremes of *Salmonella* shedding characteristics, we plotted the log transformed fecal counts at days 0, 2, 7, 14 and 20 post-inoculation for each animal. The area under the log curve (AULC) for the full 20 days was defined as the trait of interest, as this AULC is an estimate of the total *Salmonella* shed by an animal during the experiment. The number of *Salmonella* shed is clearly an important parameter that affects within-herd disease spread as well as a measure of within-animal immunologic control of *Salmonella* replication. In Figure 1, we show the accumulated AULC for each animal at each time point.

The AULC profile for *Salmonella* cfu revealed various types of *Salmonella* shedders (Figure 1 and Supplemental Table S1). For example, some pigs maintained high levels of shedding from 7 dpi to 14 dpi, gradually decreasing shedding from 14 dpi to 20 dpi and often becoming undetectable. Other pigs had high levels of *Salmonella* shedding early, which thereafter quickly decreased to low levels. We also observed pigs for which quantitative levels of *Salmonella* were only observed at 2 dpi, as well as pigs that were only qualitatively positive for *Salmonella* (Supplemental Table S1). Interestingly, from 7 dpi on, the shedding counts of several animals with low shedding levels quickly dropped close to the detection limit of 25 bacteria per gram of fecal material. However, for some animals with high initial shedding levels, significant shedding continued to 14 dpi, with some pigs even moderately increasing their shedding count during this period. These observations indicate the shedding phenotype for the LS and PS animals was manifest prior to 7 dpi. We therefore chose to compare gene expression differences at the earliest time point post inoculation, 2 dpi, between LS and PS pigs and relative to un-inoculated pigs.

The pigs were ranked by total AULC over the 20 days of challenge. Six persistent shedders (PS1) and 4 low shedders (LS1), whose ST shedding count was very high or very low, respectively, were selected for the analysis of their peripheral blood transcriptome (Figure 1 and Supplemental Table S1). Wherever possible, we selected extreme animals that had an opposite extreme littermate, to minimize genetic variation uncorrelated with shedding phenotype. Another two separate groups of pigs, designated LS2 and PS2, were selected as well for the purpose of validation of the transcriptomic results (Figure 1 and Supplementary Table S1).

### Induced large-scale gene expression changes in peripheral blood following inoculation with ST

**Figure 2:**
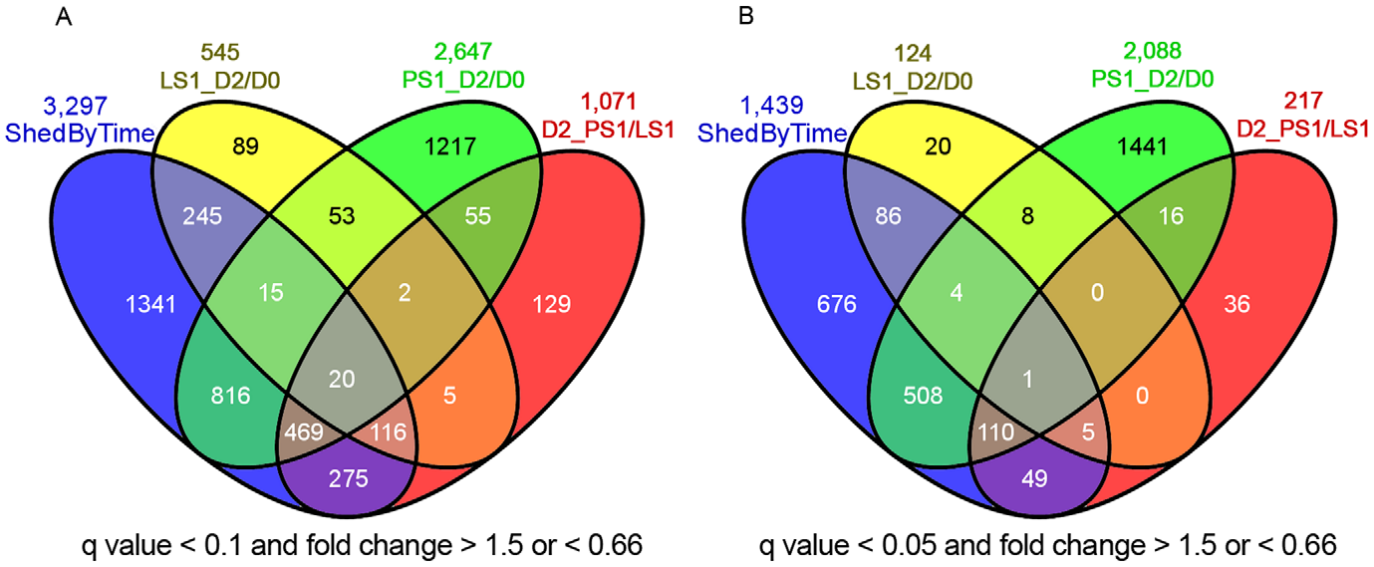
Summary of differentially expressed transcripts responding to inoculation (d2/d0), differential expression between the two shedding classes at 2 dpi (PS1/PS2), or expression showing shedding class by time interaction (Shed By Time). These genes were identified using the linear mixed model, and the false discovery rate was controlled at q less than 0.1 (Fig. 2A) or less than 0.05 (Fig. 2B), with the fold change between treatments required to be higher than 1.5 or less than 0.66 (comparing LS1 2 dpi versus 0 dpi, PS1 2 dpi versus 0 dpi, or PS1 versus LS1 at 2 dpi).

A goal of this research is to identify porcine gene expression differences that occur early during the infection that can influence the persistence and subsequent shedding of *Salmonella* in swine. Our previous investigations have demonstrated that the peak of both clinical symptoms (fever, diarrhea, decreased appetite) as well as *Salmonella* shedding occurs at 2 days post-inoculation. Thus, the peripheral blood RNA expression data for each of the 10 extreme animals (four LS1 and six PS1) at day 0 and day 2 post-inoculation were profiled and the resulting data analyzed as described in the Methods. False discovery rate (FDR) was controlled initially at 10% (q<0.1), but was reduced to 0.05% for pathway analyses after Q-PCR confirmation (see later section). The numbers of differentially expressed transcripts (probesets) for each comparison, and the numbers of transcripts that overlap among comparisons, as defined by q<0.1 and a fold change of>1.5 or <0.66 (comparing LS1 2 dpi versus 0 dpi, PS1 2 dpi versus 0 dpi, or PS1 versus LS1 at 2 dpi), are shown in Figure 2A; numbers of differentially expressed genes using a q<0.05 criterion are shown in Figure 2B. Complete data on differential expression is shown in Supplemental Table S2. Figure 2A also shows a total of 3,297 transcripts were statistically significant for tests of shedding status-by-time interaction effects. In addition, we also measured gene expression levels at day 20 for LS1 and PS1 animals. Permutation tests and traditional t-tests were used to search for expression differences between persistent shedders and low shedders (data not shown). No differentially expressed genes were identified when controlling the false discovery rate at reasonable levels (the smallest q-values were above 35%). Thus all further analyses focused on the responses at day 2.

Direct comparison before and after ST inoculation identified 545 transcripts that were differentially expressed in LS1 animals after ST inoculation and 2,647 transcripts differentially expressed in PS1 animals after ST inoculation. We also observed 1,071 transcripts differentially expressed between LS1 and PS1 animals at 2 dpi. Full lists of differentially expressed transcripts are available in Supplemental Table S3. Because changes in specific cell populations could account for these microarray results, rather than changes in cell-specific steady-state RNA levels, we tested for the correlation of cell type numbers (a standard complete blood count data including lymphocyte, monocyte, neutrophil, eosinophil, and basophil counts) with the gene expression differences. The expression of very few transcripts were correlated with cell type numbers, indicating that the gene expression differences detected by the microarray cannot be explained by changes in numbers of major cell types (Supplemental Table S4).

To initially characterize the effect of ST inoculation on canonical immune pathways such as those involved in T cell-mediated immune responses, inflammation, apoptosis, and antigen processing and presentation, we examined expression data for genes that are representative of these pathways [40]. We previously reported an increase in serum IFN-γ protein by 24–48 hours post inoculation [31]. IFN-γ-mediated activation of the cell-mediated immune response, often associated with a Th1 response, following ST inoculation was verified by observing significant increases in the RNA levels for several genes responding to IFN-γ stimulation [41], including CASP4, CD14, IL18, IRF1, IRF2, IRF7, STAT1, STAT3, OAS1, TNF, and WARS (Supplemental Table S2). Notably, IL12B-specificmRNA was not significantly affected by inoculation (IL12A is not on the Genechip), although the direction of response was opposite in LS1 versus PS1 pigs, and was close to significance for shed-by-time interaction (p<0.11; q<0.15). A classical cell-mediated activation response detected in the peripheral blood mRNA data was further demonstrated by decreases in expression levels for IL4, IL5, IL6, and IL10mRNA (Supplemental Table S2).

mRNA levels for innate/inflammatory marker genes such as SLC11A1 and TLR4 were strongly increased following ST inoculation, although IL6 and IL8 RNA levels were not affected significantly. Apoptosis pathways genes such as TGM1, CASP7, CASP8 (as well as CASP4 mentioned above) were significantly increased. Genes involved in antigen processing were up-regulated, including TAP1 and many genes encoding proteasome subunits such asPSMA1 through PSMA6, PSMB1 through PSMB10, as well as several PSMC and PSMD family members. For genes in the antigen presentation pathway, we noted that CD86 was significantly up-regulated by ST inoculation, although CD40 and CD80 were not (Supplemental Table S2). We further noted that in many of the genes mentioned above, the differential expression was observed in the PS1 but not the LS1 animals (see Supplemental Table S3 and further discussion below).

After inoculation with ST, the transcription level of more than 100 genes changed over five-fold in the PS animals (Table 1 shows the 30 genes with highest fold change; the full list is available in Supplemental Table S3). Interestingly, most of those transcripts (90%) were up-regulated. As expected, the transcripts with the largest fold change in response to ST inoculation included numerous genes that are directly connected to known immune and inflammatory responses. These include the lipopolysaccharide (LPS) receptor toll-like receptor 4 (TLR4), the S100 proteins S100A9 and S100A12 and the interleukin receptor accessory protein IL1RAP (shown in Table 1); other known immune inflammatory genes with slightly lower responses, including the arachidonate 5-lipoxygenase-activating protein ALOX5AP, the chemokine receptor CCR1, the transcription factors CEBPB and IRF7 [42], are provided in Supplementary Table S3. In addition, several genes not classically associated with immune inflammation processes were found in these highly affected genes. These include BATF3, which encodes a leucine zipper protein that functions as a transcriptional repressor, and RETN, which encodes resistin, a serum adipokine that is elevated by sepsis [43] (Table 1).

**Table 1:** Top 30 differentially expressed transcripts in PS animals with highest fold change after ST treatment (2 dpi/0 dpi), q value<0.1*.

Probeset	q Value	Fold Change	Change Direction	Gene Symbol	Similarity to RefSeq shown	Gene Description
Ssc.16234.1.S1_at	0.001	246.61	Up	TCN1	NM_001062	Transcobalamin I
Ssc.18927.1.S1_at	0.001	103.02	Up	MS4A8B	NM_031457	Membrane-spanning 4-domains subfamily A member 8B
Ssc.23801.1.S1_at	0.001	74.06	Up	RETN	NM_020415	Resistin
Ssc.12431.1.A1_at	0.002	36.06	Up	MMP8	NM_002424	Matrix metallopeptidase 8
Ssc.2381.1.A1_at	0.003	25.82	Up	S100A9	NM_002965	S100 calcium binding protein A9
Ssc.14444.3.A1_a_at	0.001	25.19	Up	ARG2	NM_001172	Arginase type II
Ssc.646.1.S1_at	0.000	19.75	Up	CSTA	NM_005213	Cystatin A
Ssc.27433.1.S1_at	0.000	18.13	Up	TGM1	NM_000359	Transglutaminase 1
Ssc.12781.1.A1_s_at	0.005	16.63	Up	TLR4	NM_138554	Toll-like receptor 4
Ssc.7864.1.A1_at	0.000	15.94	Up	IL1RAP	NM_002182	Interleukin 1 receptor accessory protein
Ssc.3556.1.A1_at	0.001	15.78	Up	-	XM_001714592	Hypothetical protein LOC100133846
Ssc.24194.1.S1_a_at	0.000	14.69	Up	TCEA3	NM_003196	Transcription elongation factor A
Ssc.3706.1.S2_at	0.000	14.52	Up	SOD2	NM_001024465	Superoxide dismutase 2 mitochondrial
Ssc.22354.1.A1_at	0.001	14.07	Up	BATF3	NM_018664	Basic leucine zipper transcription factor
Ssc.13769.1.S1_at	0.002	13.82	Up	-	XM_001127175	Hypothetical LOC728320
Ssc.3012.1.S1_at	0.004	13.32	Up	UPP1	NM_181597	Uridine phosphorylase 1
Ssc.17283.2.S1_at	0.012	12.09	Up	-	NM_001038000	FKBP1A-like
Ssc.15379.1.S1_at	0.000	11.82	Up	DGAT2	NM_032564	Diacylglycerol O-acyltransferase homolog 2
Ssc.14533.1.S1_at	0.002	11.66	Up	-	NM_002910	Renin binding protein
Ssc.998.1.A1_at	0.001	10.71	Up	FAM129A	NM_052966	Family with sequence similarity 129 member A (FAM129A)
Ssc.30887.1.S1_at	0.017	10.58	Up	TNFAIP6	NM_007115	Tumor necrosis factor alpha-induced protein 6
Ssc.1137.1.S1_at	0.003	10.57	Up	CASZ1	NM_001079843	Castor zinc finger 1
Ssc.2697.1.S1_at	0.002	10.53	Up	TCEA3	NM_003196	Transcription elongation factor A
Ssc.5053.1.S1_at	0.001	9.33	Up	CD163	NM_203416	CD163 molecule
Ssc.25255.1.S1_at	0.001	9.04	Up	ASRGL1	NM_001083926	Asparaginase like 1
Ssc.7839.1.A1_at	0.018	9.02	Up	EPB41L3	NM_012307	Erythrocyte membrane protein band 4.1-like 3
Ssc.9117.1.S1_at	0.002	9.01	Up	S100A12	NM_005621	S100 calcium binding protein A12
Ssc.15890.1.S1_at	0.002	12.50	Down	VNN1	NM_004666	Vanin 1
Ssc.6943.1.A1_at	0.027	9.09	Down	ANGPT1	NM_001146	Angiopoietin 1
Ssc.19586.1.S1_at	0.002	9.09	Down	C1orf210	NM_182517	Chromosome 1 open reading frame 210

*For multiple probesets that had the same annotation, the probeset with highest estimated expression level was retained.

Two of the four top-ranked transcripts, which are annotated as TCN1 and MMP8, encode proteins that are major constituents of secondary granules in neutrophils [44], [45], were up regulated 246- and 36-fold, respectively. Level of TCN1 RNA has been reported as very low in blood from healthy humans (http://biogps.org/#goto=genereport&id=6947), and our results indicate expression is high only after innate immune responses are initiated. Such regulation of TCN1 is supported by a report showing induction of TCN1 RNA in the intestinal epithelium of cholera patients compared to healthy controls [42]. The transcripts for S100A9 and S100A12 were up-regulated 25.8- and 9-fold, respectively. Alteration of the expression of these two S100 protein genes has been reported to be associated with neutrophil functions and the onset of disease [46]. Thus multiple high-ranked (significant and high fold change) differentially expressed genes were associated with neutrophils, indicating that neutrophils play an important role in the PS responses to ST inoculation. The non-significant correlation of the neutrophil counts with the gene expression differences indicated that the demonstrated increase in specific transcripts for these genes was not caused by higher numbers of circulating neutrophils but by increased gene expression in the blood.

Compared with the PS1 animals, the transcriptomic responses of LS1 animals were significantly less dramatic; only 12 genes show |fold change|>3 and q<0.1 (Table 2 shows the genes with |fold change|>3; the full list is available in Supplemental Table S3). For all 124 differentially expressed genes (q<0.1), 13 were also differentially expressed in PS1animals. Interestingly, four of these genes were changed in the opposite direction between LS1 and PS1 groups. The gene up-regulated most dramatically, TGM1, has been reported to be significantly induced by SC, ST, and *Haemophilus parasuis* [26], [40], [47], [48]. Other top-ranked differentially expressed genes in the LS1 pigs such as KRTAP11-1, TAF1B and BTBD10, have not been associated with *Salmonella* infection previously.

**Table 2:** Top ranked differentially expressed transcripts in LS after ST treatment (2 dpi/0 dpi), q value<0.1, | fold change |>3.0*.

Probeset	q Value	Fold Change	Change Direction	Gene Symbol	Similarity to RefSeq shown	Gene description
Ssc.27433.1.S1_at	0.050	6.51	Up	TGM1	NM_000359	Transglutaminase 1
Ssc.2131.1.S1_at	0.048	6.15	Up	TMTC1	NM_175861	Transmembrane and tetratricopeptide repeat containing 1
Ssc.15379.1.S1_at	0.049	5.53	Up	DGAT2	NM_032564	Diacylglycerol O-acyltransferase homolog 2
Ssc.18987.2.A1_at	0.039	4.30	Up	GPN2	NM_001031770	Bos taurus GPN-loop GTPase 2
Ssc.25697.1.S1_at	0.046	3.83	Up	KRTAP11-1	NM_175858	Keratin associated protein 11-1
Ssc.22089.2.S1_at	0.049	3.78	Up	TAF1B	NM_005680	TATA box binding protein (TBP)-associated factor
Ssc.2798.1.S1_at	0.031	3.02	Up	SYP	NM_003179	Synaptophysin (SYP)
Ssc.420.4.S1_a_at	0.038	3.23	Down	CAMP	NM_004345	Cathelicidin antimicrobial peptide
Ssc.30090.1.A1_at	0.046	4.35	Down	GPN2	NM_001031770	GPN-loop GTPase 2
Ssc.26110.1.S1_at	0.049	5.56	Down	BTBD10	NM_032320	BTB (POZ) domain containing 10
Ssc.7361.1.A1_at	0.031	8.33	Down	-	-	-
Ssc.12131.1.A1_at	0.046	10.00	Down	-	-	-

*For multiple probesets that had the same annotation, the probeset with highest estimated expression level was retained.

The transcriptional response in the LS1 and PS1 animals is quite distinct; most of the genes in either LS1_D2/D0 or PS1_D2/D0 were not found in the differentially expressed list for the other class; only 13 genes were in common between these two sets of genes (Figure 2B). In addition, 217 genes were significantly differentially expressed between LS1 and PS1 pigs at day 2 after ST inoculation (Figure 2B). Of these, 127 were also differentially expressed in PS1 animals in response to infection (those genes found in both D2_PS1/LS1 and PS1_D2/D0, Figure 2B), and all of these were up regulated (higher in post-inoculation samples of PS1 animals). Six of the 217D2_PS1/LS1 genes were also changed in the LS1 animals after ST inoculation (LS1_D2/D0) and five of them were up regulated. Of these six, only one is also differentially expressed in PS animals.

Many genes were dramatically differentially expressed between the PS1 and LS1 phenotypic classes (Table 3 shows the genes with |fold change|>3, the full list is available in Supplemental Table S3). Such genes include the MS4A8B gene, which increased 103-fold in PS animals after ST treatment and was not significantly different in LS animals. It was reported that MS4A8B was significantly induced by *Haemophilus parasuis*, indicating its relationship with bacterial infections in pigs [47]. The CSTA, BTBD10, KIF1B, RARS2, and HPSE were expressed over 5 fold or higher in PS over LS animals. The CD55 gene, which encodes a protein involved in the disruption of the complement cascade, was 4.6 fold higher in PS over LS animals. The C1orf210, PSP-II, GPN2, and SEPT8 genes were expressed over 5 fold lower in PS animals compared to LS animals. Expression differences between the shedding phenotypes were also observed for other genes such as C1 peptidase (CTSS), which participates in the degradation of antigenic proteins to peptides for presentation on MHC class II molecules; MD-2 (LY96), whose protein associates with TLR4and provides a link between the receptor and downstream LPS signaling. An increase in the mRNA for the Toll/interleukin-1 receptor domain-containing adaptor protein, TICAM2, was also statistically significant (q value<0.05 and fold change>1.5) (Supplemental Table S3).

**Table 3:** Top ranked differentially expressed probesets between PS and LS animals after ST treatment at 2 dpi, q value<0.1, | fold change |>3.0*.

Probeset	q Value	Fold Change	Change Direction	Gene Symbol	Similarity to RefSeq shown	Gene description
Ssc.18927.1.S1_at	0.038	27.04	Up	MS4A8B	NM_031457	Membrane-spanning 4-domains, subfamily A, member 8B (MS4A8B)
Ssc.646.1.S1_at	0.024	8.69	Up	CSTA	NM_005213	Cystatin A
Ssc.26110.1.S1_at	0.018	7.07	Up	BTBD10	NM_032320	BTB (POZ) domain containing 10
Ssc.25195.1.A1_at	0.043	6.34	Up	KIF1B	NM_183416	Kinesin family member 1B
Ssc.10706.1.A1_at	0.045	6.21	Up	RARS2	NM_020320	Arginyl-tRNA synthetase 2 mitochondrial
Ssc.7093.3.S1_at	0.034	6.10	Up	HPSE	NM_001098540	Heparanase (HPSE)
Ssc.22694.1.S1_at	0.045	5.72	Up	NDUFB6	NM_182739	NADH dehydrogenase (ubiquinone) 1 beta subcomplex 6
Ssc.5618.1.S1_at	0.043	5.40	Up	RALGDS	NM_001042368	Ral guanine nucleotide dissociation stimulator
Ssc.2697.1.S1_at	0.044	5.28	Up	TCEA3	NM_003196	Transcription elongation factor A
Ssc.21060.1.A1_at	0.044	5.07	Up	Golim4	NM_175193	Golgi integral membrane protein 4
Ssc.19389.1.A1_at	0.034	4.87	Up	C15orf48	NM_032413	Chromosome 15 open reading frame 48
Ssc.271.1.A1_at	0.045	4.66	Up	CD55	NM_001114752	CD55 molecule decay accelerating factor for complement
Ssc.24277.1.S1_at	0.047	4.25	Up	USP45	NM_001080481	Ubiquitin specific peptidase 45
Ssc.11382.1.S1_at	0.040	4.18	Up	SERINC1	NM_020755	Serine incorporator 1
Ssc.29675.1.S1_at	0.038	4.18	Up	RABL3	NM_173825	member of RAS oncogene family-like 3 (RABL3)
Ssc.19596.2.S1_at	0.045	4.06	Up	Lpcat2	NM_173014	Lysophosphatidylcholine acyltransferase 2
Ssc.13053.1.A1_at	0.047	4.05	Up	CASP4	NM_176638	Apoptosis-related cysteine peptidase
Ssc.12711.1.S1_at	0.045	3.70	Up	SSFA2	NM_001130445	Sperm specific antigen 2
Ssc.25255.2.S1_a_at	0.045	3.47	Up	ASRGL1	NM_001083926	Asparaginase like 1
Ssc.8473.1.S2_at	0.034	3.40	Up	DNAJA1	NM_001539	DnaJ (Hsp40) homolog subfamily A member 1
Ssc.22158.1.S1_at	0.038	3.33	Up	ABHD13	NM_032859	Abhydrolase domain containing 13
Ssc.3706.1.S1_at	0.038	3.20	Up	SOD2	NM_001024465	Superoxide dismutase 2
Ssc.5190.1.S1_at	0.034	3.06	Up	HIATL1	NM_032558	Hippocampus abundant transcript-like 1
Ssc.6798.2.S1_at	0.038	3.04	Up	HSPA9	NM_004134	Heat shock 70 kDa protein 9
Ssc.12446.1.A1_at	0.046	3.04	Up	CASP4	NM_033306	Apoptosis-related cysteine peptidase
Ssc.6618.1.A1_at	0.038	3.33	Down	ENDOD1	NM_015036	Endonuclease domain containing 1
Ssc.16377.2.A1_at	0.034	3.70	Down		NM_145740	Glutathione S-transferase A1
Ssc.3344.1.A1_at	0.043	5.56	Down	SEPT8	NM_015146	Septin 8
Ssc.18260.1.A1_at	0.038	5.56	Down	GPN2	NM_001031770	GPN-loop GTPase 2
Ssc.590.1.S1_at	0.043	5.56	Down	PSP-II	NM_213836	Porcine seminal protein II (PSP-II)
Ssc.19586.1.S1_at	0.044	8.33	Down	C1orf210	NM_182517	Chromosome 1 open reading frame 210

*For multiple probesets that had the same annotation, the probeset with highest estimated expression level was retained.

### Validation of differentially expressed genes using qPCR

**Figure 3:**
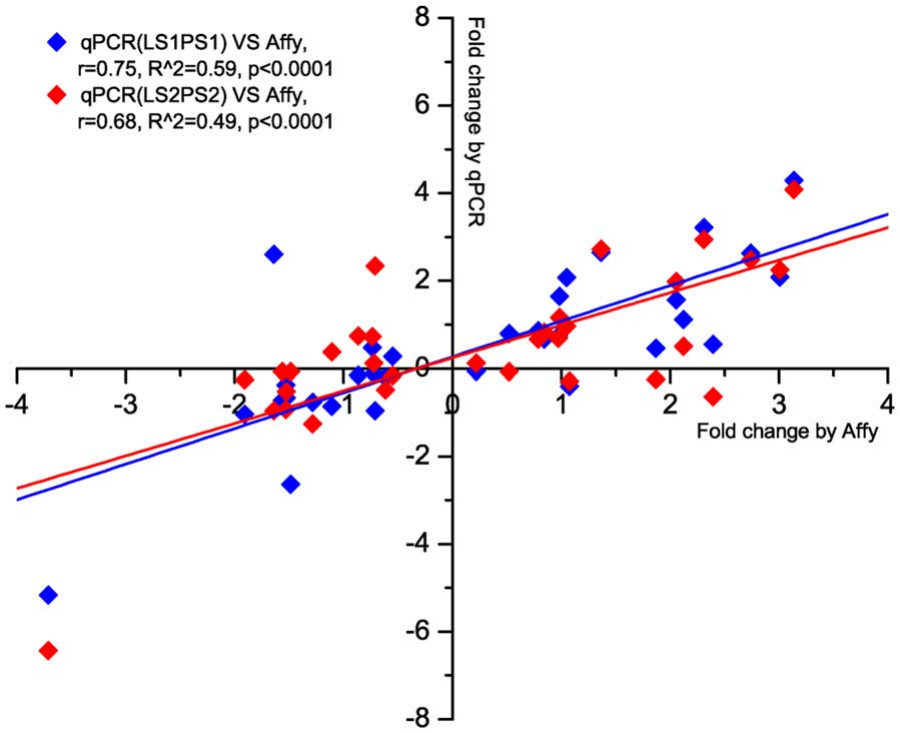
Significant correlation between qPCR and microarray expression measurements across 21 genes tested. Whole blood gene expression levels of the LS1, PS1 and LS2, PS2 pigs detected by qPCR were compared with microarray results for LS1 and PS1 samples. The diamonds indicate the fold changes for each of the comparisons (LS_D2/D0, PS_D2/D0, D2_PS/LS), as measured using Affymetrix technology (X-axis) or by Q-PCR (Y-axis). The lines illustrate the linear regression between expression levels detected by the two different methods.

Twenty-one differentially expressed genes were selected for validation of the microarray data by using qPCR (Table 4). All raw data and statistical results of these qPCR tests are available in Supplemental Table S5. The genes were selected with respect to their rank of expression changes (q<0.1, fold change>1.5 or <0.66), biological function, quality of annotation, available nucleotide sequence length, and estimated microarray signal intensity as an indicator of transcript abundance. Correlation analysis demonstrated that, overall, the qPCR results were significantly correlated with the microarray results, indicating that the qPCR results generally confirmed the microarray gene expression data (Figure 3, r = 0.75, p<0.0001). For 15 selected genes detected by microarray as differentially expressed in PS animals after ST inoculation, 10 of them were also significant (p<0.05) or trended to significance (p<0.1) by qPCR (Table 4). For the remaining six genes, five of them were changed in the same direction as the microarray data. For the nine genes differentially expressed between PS and LS animals detected by microarray, four of them were confirmed by qPCR (p value<0.1), and four out of the remaining five genes were changed in the same direction by both techniques. For the six genes differentially expressed in LS animals after ST inoculation, one gene tended to significance in the qPCR data (p value = 0.12), and three out of the remaining five genes, were changed in the same direction in both microarray and qPCR results. These results indicated that while the qPCR and microarray results were generally in agreement, less dramatic expression differences in LS animals were difficult to confirm by qPCR. We also observed that genes with more statistically significant differences in the microarray analysis (q value<0.05) were nearly always confirmed by qPCR (95% consistency), while it was more difficult to validate genes with higher q values (69% consistent for 0.05<q<0.1; Table 4).

**Table 4:** qPCR results for gene expression in pigs with different shedding phenotypes at day 0 versus day 2 following ST inoculation, comparing with microarray data.

	Microarray data (FC^a^)			qPCR for group #1 (FC^a^)			qPCR for group #2 (FC^a^)		
Gene Name	D2 PS/LS	LS D2/D0	PS D2/D0	D2 PS/LS	LS D2/D0	PS D2/D0	D2 PS/LS	LS D2/D0	PS D2/D0
RPL6	0.60#	1.16	0.68	0.92	0.96	1.21	1.08	1.08	0.89
CEBPB	2.24	2.68	8.03##	3.55*	0.42	4.24*	1.59	3.89**	4.74**
CASP1	2.07#	1.74	4.15##	4.20**	0.43	2.95*	1.94**	4.16**	3.95**
SLC11A1	2.23	2.45	8.78##	4.55**	2.81	19.6**	4.03**	4.40**	16.9**
TLR4	1.81	2.23	4.96##	3.34*	4.26*	9.30**	1.19	5.22**	7.68**
VNN1	0.24	0.28	0.07##	0.02**	0.71	0.02**	0.09	0.01**	0.01*
TLR2	1.79	2.10#	3.65##	1.69	0.75	1.37	1.56	0.82	0.84
IGHG	0.34##	0.86	0.41##	0.63*	0.93	0.58**	0.69	0.79	0.41**
TREM1	1.86	2.07	5.26##	2.47*	0.17*	1.46	1.07	0.84	0.64
BPGM	0.34##	1.16	0.33##	0.77	1.13	0.60	0.52	1.46	0.95
ARPC4	1.79#	0.90	1.97##	1.59*	1.09	3.13**	1.77**	3.10**	2.24**
IRF7	1.06	2.37	6.67##	1.27	1.90	6.21**	1.83	2.45	5.55*
SPI1	1.48	1.95	2.57##	1.84	3.24**	6.28**	1.45	3.78**	6.61**
C1QA	0.32#	2.29	0.35##	6.06*	0.01**	0.16	0.51	1.40	0.95
LEF1	1.20	0.69	0.65##	1.74	1.02	0.87	1.12	0.71	0.71
HSP90AA1	1.72#	0.83	1.43	1.82*	0.64	1.73*	1.58*	1.09	0.95
HSPD1	1.70	0.61#	1.23	3.28*	0.51	2.28	0.96	5.06**	3.44**
NCL	4.35#	0.26#	1.76#	2.16	0.48	1.22	1.41	0.83	0.83
ZFP36L2	1.44	0.59#	0.87	1.41	1.38	1.55	1.27	1.65	1.38
SFRS3	1.60	0.46##	1.06	1.51	0.55	1.02	1.86	1.29	1.88
DDX3X	1.96#	0.54#	1.05	1.66	0.90	1.29	1.61	1.67	1.41

a FC, fold change: the ratio of the Ct value (qPCR) or probe intensity (microarray) between contrasts. FC greater than 1.0 indicates the gene was up-regulated, FC less than 1.0 indicates the gene was down-regulated for the two samples shown.

*Statistical test of difference between contrasts: *indicates trends to significance at p<0.1 (Student's t-test), **indicates significant difference at p<0.05.

# All genes shown were selected for qPCR validation. The genes were selected with respect to their rank of expression changes (^#^q<0.1, fold change>1.5 or <0.66; ^##^q<0.05, fold change>1.5 or <0.66;), as well as biological function, quality of annotation, available nucleotide sequence length, and estimated microarray signal intensity as an indicator of transcript abundance.

Expression levels of the selected genes in 10 additional animals (four PS2 and six LS2) were measured by qPCR for further validation of the gene expression differences detected by microarray. The qPCR results again in the aggregate correlated significantly with the microarray results (Figure 3, r = 0.68, q<0.0001), indicating that differentially expressed genes detected by microarray can be confirmed by qPCR even in a new group of pigs. Altogether, 40 samples have been measured by qPCR (20 animals, 2 time points), with a total of 84 comparisons made (LS_D2/D0, PS_D2/D0, D0_PS/LS, D2_PS/LS; Table 4). Of these, 11 comparisons (13%) with q value<0.1 (by microarray), showed a response in the opposite direction between qPCR and microarray results. Overall, 85% of the genes with the most significant differences in expression (q value less than 0.05 and fold changes higher than 1.5 or greater than 0.66) showed consistency between microarray and QPCR methods. Therefore, to be conservative in all further analyses, differentially expressed genes were defined using a threshold of q value<0.05 and fold-change greater than 1.5 or less than 0.66.

### Functional annotation of the differentially expressed genes and clusters of co-expressed genes

The functions of the differentially expressed genes and the possible relationship between ST inoculation and the alteration of gene expression were investigated by using DAVID [39], and InnateDB [49]. The DAVID database was used to identify over-represented functional GO terms for differentially expressed genes (q<0.05, Supplemental Table S6). For the genes up-regulated in PS animals following ST inoculation, 262 genes were linked to 38 significantly over-represented GO terms. The seven top ranked GO terms related to immune and inflammatory response” or “defense response” covered 106 genes (about 40% of the total), indicating that these terms represent the major responses in the ST-inoculated PS animals. The GO term of “proteasome”, which consists of 14 proteasome subunits, was significantly over-represented as well; this annotation is consistent with the PSMA-D genes noted above. For the down-regulated genes in PS animals, only six GO terms reached the significance level. However, the terms were very general (i.e., “glycoprotein”, “secreted” and “signal”) and the specific biological meaning of these differentially regulated genes is unclear.

For the up-regulated genes in the LS animals following ST inoculation, none of the GO terms reached the significant level of 0.05 for over-representation, although a large number of genes were connected to GO terms such as “immune response” and “inflammatory response”. For the down-regulated genes in the LS1 animals, 17 GO terms were significantly over-represented and most of these GO terms are related to RNA binding and recognition. There were 111 genes identified that are differentially expressed in the LS1 animals only and not in the PS1 animals (86+20+5, Figure 2B). Such genes are of interest since they are associated with a decrease in *Salmonella* shedding in the LS1 animals. Interestingly, most of these genes were down-regulated (104 out of 111) in LS1 compared to PS1 pigs following ST inoculation, and GO annotation analysis of these down-regulated genes revealed their involvement in “mRNA processing/metabolic process,” “nucleotide binding,” “response to stress or DNA damage stimulus,” and “cell cycle related process” (Supplemental Table S6). For the genes expressed higher in the PS1 over LS1 animals at 2 dpi, only two GO terms were significantly over-represented, “endosome” and “Golgi apparatus”. For the genes expressed higher in LS1 over PS1 animals, a large number of genes were connected with GO terms such as “ribosome” or “translation regulation”. While these annotations were non-significant, they were close to the q<0.05 significant levels for over-representation.

**Figure 4:**
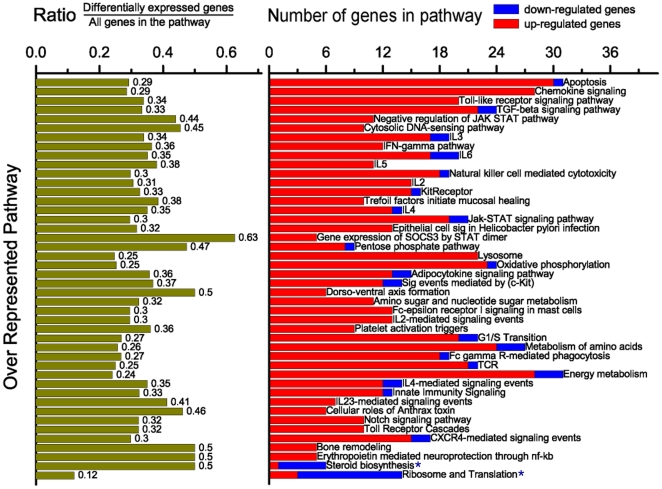
Over-representation of many InnateDB pathways in the differentially expressed transcript list of ST inoculated pigs. The lists of differentially expressed genes in PS1 pigs (q<0.05) were annotated using DAVID [39] and over-represented pathways identified using a false discovery rate of q<0.1 in InnateDB [38]. Left: the ratio represents the proportion of the differentially expressed genes in a specific pathway compared to all of the genes in that pathway. Right: each row represents the counts of the differentially expressed transcripts similarly annotated as shown by the row label. As calculated by InnateDB, most of the genes in a majority of the pathways shown are up-regulated (q<0.1); however, the bottom-most two pathways (marked with an *) are enriched with down-regulated genes (q<0.1).

**Figure 5:**
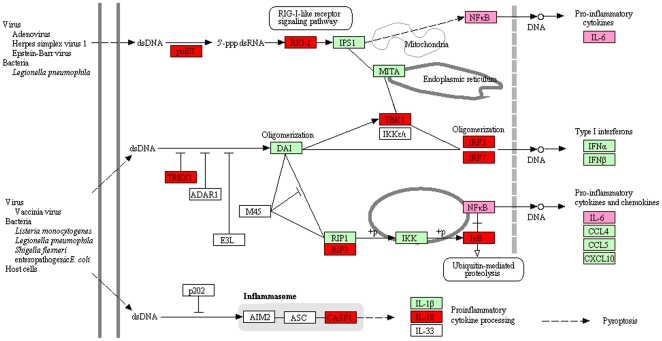
Genes in the Cytosolic DNA-sensing pathway show changes in expression during response to Salmonella. The red boxes indicate up-regulated genes and pink boxes indicate the gene is up-regulated and that target genes of the gene in the pink box are over-represented in DAVID analysis. Green boxes indicate the gene is present on the array but was not differentially expressed. White boxes indicate that no gene information was available in the porcine genome, or that the gene was not on the Affymetrix Genechip.

To further analyze the differentially expressed gene lists, pathway over-representation analysis was performed using the online tool, InnateDB, which can identify the major interconnected innate immunity networks in response to ST inoculation (Figure 4 and Supplemental Table S7). Forty-three pathways were significantly over-represented (q<0.1), with 42 of them up-regulated. As observed for the GO annotation analysis, most of these over-represented pathways (40 of 43) derive from the genes differentially expressed in the PS1 animals in their response to *Salmonella*. The up-regulated pathways included those required for inflammatory and immune responses, such as “Apoptosis,” “Chemokine signaling,” “Toll-like receptor signaling,” “IFN-γ,” “interleukin signaling,” “Jak-STAT signaling pathway” and “Fc-γ receptor-mediated phagocytosis” (Figure 4). The “Apoptosis pathway,” “Chemokine signaling pathway,” “Integration of energy metabolism,” “Metabolism of amino acids” and “Oxidative phosphorylation” were the top five pathways with the highest number of up-regulated genes, and possibly represent the major biological changes in the blood of PS1 animals after ST inoculation. The “Cytosolic DNA-sensing pathway,” which could be another important regulatory module responsible for the up-regulation of the genes in the PS1 animals after ST inoculation, was also significantly over-represented (Figures 4 and 5). Interestingly, some of the pathways enriched with up-regulated genes such as “Apoptosis pathway,” “IL3 pathway,” and “Jak-STAT signaling pathway” also included a few genes that were down-regulated, indicating the complexity of the internal connections and regulation of these pathways. The “Steroid biosynthesis pathway” was significantly down-regulated after ST inoculation in PS1 animals. For the lists of genes differentially expressed between PS1 and LS1 animals at 2 dpi, two pathways, “Steroid Biosynthesis” and “Ribosome and Translation pathway” were significantly over-represented in genes with lower expression in LS1 animals. Finally, no Innate DB annotated pathway was significantly over-represented in LS1 animals due to ST inoculation.

### Identifying sets of co-regulated genes and their differentially expressed regulators in response to ST inoculation

**Figure 6:**
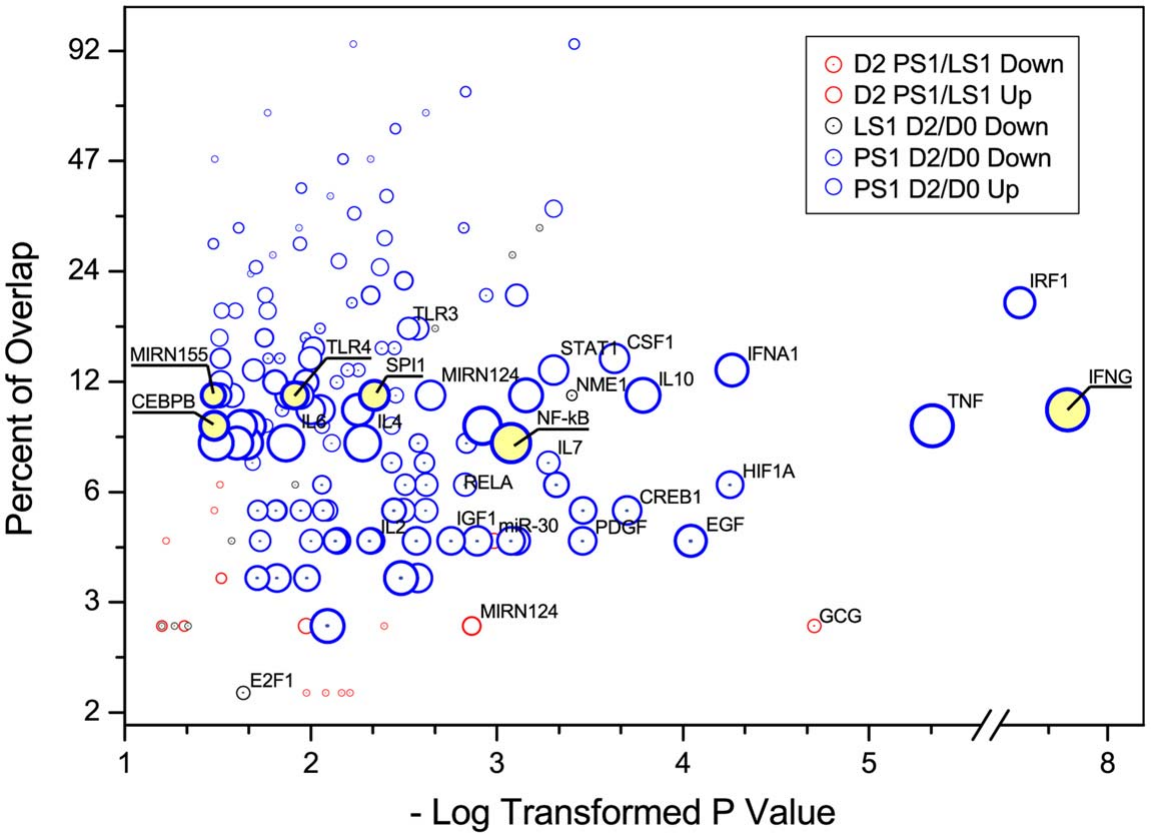
Many sets of transcripts whose expression is controlled by a common regulator (within the same regulon) are found significantly enriched in the differentially expressed transcript lists. Differentially expressed transcripts for a particular comparison (PS1 2 dpi versus 0 dpi, etc.) are shown with specific colors. The x axis is the negative log of the p value for the over-representation of the regulon shown. The y axis (percent overlap) represents the proportion of the differentially expressed targets by a specific expression regulator compared to all known targets of that regulator. The size of the bubble reflects the number of differentially expressed targets for each regulon. For all regulons described in the text, the bubble is underlined.

Using regulator-target relationships known from PubMed literature, we used Sub-Network Enrichment Analysis (SNEA) to identify individual “regulators” whose connections to specific sets of differentially expressed target genes were over-represented (p<0.05; Figure 6 and Supplemental Table S8). Such relationships include direct interactions as well as more indirect relationships inferred from published sources. Very few common GO annotations of target genes were significantly over-represented in the lists of differentially expressed genes in LS1 animals due to ST inoculation and between PS1 and LS animals at 2 dpi. In the response to *Salmonella* in PS1 pigs, however, many regulator-target relationships were statistically significant, including those involving cytokines such as IFN-γ and tumor necrosis factor (TNF) and to immune-inflammatory related transcription factors such as NF-κB. IFN-γ, TNF, and NF-κB were linked to the largest number of differentially expressed genes, indicating their important roles in the regulation of gene expression responses due to ST infection. Three interleukin subnetworks (genes regulated by IL4, IL10 or IL12) were also significantly enriched. These interleukins have extensive effects on T cell activation, B cell proliferation, natural killer cell activation, and antibody production, and also have broad interactions with other immune and inflammatory modulators such as IFN-γ and NF-κB. Several other genes with altered expression patterns following ST inoculation, including IRFs and STATs, as well as SPI1, CEBPB, RELA and TLR4, had significantly enriched regulator-target sets (Figure 6 and Supplemental Table S8). Four regulators with significantly enriched down-regulated targets,JUN, MAPK14, RELA and NR1H3, were induced in response to ST challenge and two such regulators (FOS and FOXO3) were repressed. The target network of TGFB1, whose protein product acts as a negative autocrine growth factor, was significantly enriched as well. The relevant functions of other over-represented regulators and subnetworks of regulated targets are less defined.

**Figure 7:**
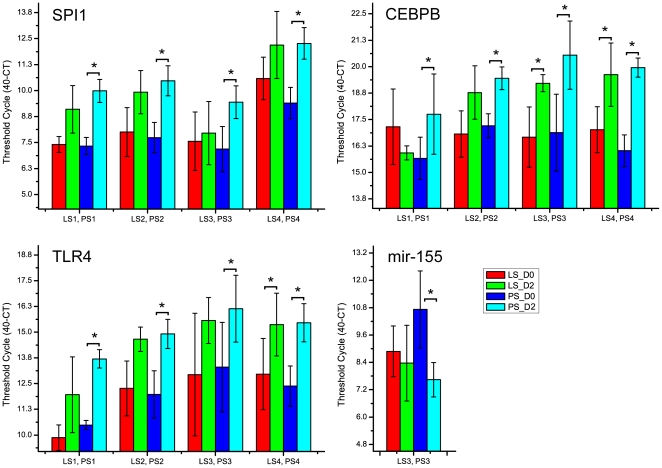
Reciprocal expression during the persistent shedder response to *Salmonella* inoculation for miR-155 and two of its repressed targets, SPI1 and CEBPB, which themselves have over-represented target gene sets in the transcriptomic response to *Salmonella*. Expression level is presented as 40-minus actual C_t_. LS1PS1, LS2PS2, LS3PS3, LS4PS4 are four different sets of low shedding and persistent shedding pigs selected from two different challenge populations. The miR155 data was collected only from LS3PS3 due to the fact that sufficient samples for the other pigs for miRNA preparations were unavailable.

Differential expression in PS animals for three regulators with over-represented targets, SPI1, CEBPB and TLR4, was confirmed by qPCR (Figure 7). All three were significantly up-regulated at 2 dpi in all four sets of the PS animals (PS1 to PS4) after ST inoculation. The expression levels of two miRNAs (mir-124 and mir-155) with over-represented targets were quantified by Stem-loop TaqMan Assay (Figure 7). While the expression of mir-124 in peripheral blood was almost undetectable (data not shown), miR-155 was down regulated in pig peripheral blood RNA from PS3 animals inoculated with ST (over 5 fold, p<0.01, Figure 7).

## Discussion

### Blood transcriptome analysis as a means to explore innate immune responses and identify biomarkers for variation in disease outcome

The blood transcriptome has emerged as a useful and practical window into the status of the immune system in health and disease [27], [50], even for compartmentalized infections such as respiratory disease [28], [51]. Additionally, variation in immune response to the same pathogen among individuals can be detected; a recent report described blood transcriptional signatures that correlated with the extent of pulmonary tuberculosis [51]. To date, there have been only a few reports of the response of the porcine whole blood transcriptome to infection [35], [52]. In contrast, several studies have assessed the responses of peripheral blood derived cell populations to infection with bacteria or viruses [53], [54], [55], [56], [57], immune stimulants [58], or following vaccination [59]. Several reports have demonstrated there is genetic control of various immune cell parameters or immunological traits [8], [9], [10], [12], [13], [14], as well as loci associated with susceptibility to salmonellosis [15], [16]. Our focus in this work was to identify genes that mark early differential immune responses to *Salmonella* in pigs by correlating fecal shedding and blood transcriptomic responses. The expression changes of such early biomarker genes associated with shedding could potentially be used to select animals that are less susceptible to persistent infection, and thus serve as a useful phenotype to improve pig genetics and food safety problems introduced by *Salmonella* infection. In future work, it is also possible to search for genetic polymorphisms controlling the variation of these expression patterns, using the candidate gene approach (Uthe et al., in press) or an expression Quantitative Trait Loci (eQTL) approach [60]. Analysis of whole blood mRNA expression would also be very valuable as a low cost means for measurement of genes whose expression patterns are associated with a superior outcome, so that the large numbers of individuals required for such association studies can be tested and any association measured.

### Identifying blood transcriptome patterns associated with fecal shedding of *Salmonella* Typhimurium

Enumeration of fecal *Salmonella* shedding across the challenge populations indicated that substantial shedding variability exists among individual pigs. The transcriptome profiling results demonstrated that the PS1 animals, which shed significantly higher amounts of *Salmonella* than the LS1 animals, also have significantly more vigorous peripheral blood transcriptome responses. Further, these results indicated the induction of a cell-mediated immune response with expression of genes classically associated with a Th1 response and typified by IFN-γ signaling pathways; these pathways are discussed in detail below.

The GO annotation analysis of genes responding to *Salmonella* colonization at 2 dpi indicated that the innate immune genes constituted the majority of the over-represented responses and this was confirmed by InnateDB analysis. Furthermore, InnateDB over-representation analysis identified that the TLR4 and IFN-γ systems are major inducers of transcriptomic responses in the peripheral blood of PS1 animals. The significant up-regulation of mRNA for TLR4 and the elevation of over 30% of the genes in the TLR signaling pathway indicated that this pathway and the downstream effectors were extensively activated early in the response to *Salmonella*. The activated downstream cascades of the chemokine signaling pathway, the “NF-κB induced response,” the “IFN-γ cascade,” and the interleukin pathways together with the “Toll-like receptor signaling pathway” itself cover most of the over-represented pathways, further indicating primary roles for the TLR4 and IFN-γ systems in this response.

The cytosolic DNA-sensing pathway, which was significantly up-regulated in the PS animals, could be another important regulatory module responsible for the induced expression patterns seen in the PS1 animals after ST inoculation. This pathway includes specific families of pattern recognition receptors responsible for the detection of foreign DNA from invading microbes and for generating innate immune responses, such as the double-stranded DNA sensor protein DAI and AIM2 [61], [62], [63]. Foreign DNA can also be converted to RNA by host RNA polymerase III and then recognized by the RNA sensor RIG-I [62], [63]. After ST inoculation, 45% of genes in this pathway were significantly induced, including the RNA polymerase III gene POLR3GL, the DNA exonuclease TREX1, and the double-stranded RNA recognition protein DDX58, all of which are very important for the function of the Cytosolic DNA-sensing pathway (Figure 4 and 5). Most importantly, the signaling modules downstream of the cytosolic DNA-sensing pathway such as the NFκB-, IL6-, and IFNα-, centered cascades were also significantly changed after ST inoculation (Figure 4 and 5). These results indicate that the cytosolic DNA-sensing system may be an important mechanism for the activation of the immune and inflammatory responses in persistently shedding pigs.

### Decreases in expression of genes involved with RNA metabolism and splicing are associated with low shedding phenotype

A clearly intriguing result is the group of 111 genes, mostly down-regulated upon inoculation with *Salmonella*, that were identified as differentially expressed in the LS1 animals (Figure 2B), and thus are associated with reduced *Salmonella* shedding and a potentially superior host control of *Salmonella* replication. Many of the over-represented terms in this set of genes are related to RNA processing, metabolism, and/or splicing. Alternative splicing plays an important role in several aspects of host-pathogen interactions and immunity, and many examples of alternative splicing of RNAs encoding immune system components have been reported [64], [65], [66], [67], [68]. Two genes SFRS1 and SFRS3, members of the serine-arginine-rich (SR) family are directly related to alternative splicing. SFRS1 and SFRS3 were down-regulated at 2 dpi specifically in the LS1 animals. Both SFRS1 and SFRS3 contribute to viral RNA nuclear export [69], [70], and also interact with viral proteins to regulate protein production during several viral infections [71], [72], [73], [74]. Therefore, decreasing SR gene expression could be postulated as a useful response to viral infection. It would be quite interesting to explore the role of SFRS1 and SFRS3 in *Salmonella* colonization, as the decreased expression of these genes at day 2 pi is correlated with lower *Salmonella* shedding observed in the LS animals.

We were also interested in exploring the mechanisms by which expression of splicing factor genes could be decreased. Beta-catenin signaling can activate expression of SFRS3 thus affecting alternative splicing [75]. Expression of CTNNB1, the gene encoding β-catenin that controls expression of target genes through its interaction with TCF4, is decreased significantly in LS1 animals after *Salmonella* inoculation (1.6 fold relative to day 0, q<0.04) and is slightly increased in PS1 pigs (1.2 fold, q<0.07). Thus, the decrease of SFRS3, and potentially alternative splicing in general, could be due to decreased β-catenin signaling in the LS1 pigs. Interestingly, *Salmonella* infection of mice was shown to stimulate β-catenin protein degradation, decrease expression of c-myc (aβ-catenin target gene), and decrease the physical interaction between β-catenin and NF-κB [76]. Further, constitutively active β-catenin was shown to stabilize the NF-κB inhibitor I-κB, indicating β-catenin may have a novel role in suppression of inflammation induced by *Salmonella* or other pathogenic bacteria [77]. In addition, *Salmonella* effector protein AvrA was recently shown to increase β-catenin protein levels, leading to activation of the Wnt/β-catenin pathway signaling in intestinal epithelial cells [78]. Finally, it has recently been demonstrated that β-catenin in gut dendritic cells is necessary for anti-inflammatory responses, and β-catenin signaling is thought to promote tolerance to inflammatory stimuli [79]. We observed specifically in the LS1 pigs a decreased expression of β-catenin and targets of β-catenin that are known to control alternative splicing of immune response genes. It would be of interest to test whether effects on alternative splicing are associated with decreased shedding after *Salmonella* inoculation.

### Multiple annotation analyses indicate TLR and IFN-γ regulons can account for much of the immune response pathways that are associated with animals with the highest levels of *Salmonella* shedding

The TLR and IFN-γ signaling pathways are prominent in the PS1 response to *Salmonella* inoculation, as shown by both the GO and InnateDB analyses. We also used SNEA to identify regulatory proteins linked to a network (regulons) of their known targets that are enriched among the members of the differentially expressed gene lists. If network regulators are themselves differentially expressed, this could provide evidence for specific hypotheses that such regulators are responsible for the observed changes in expression of their enriched target genes. For example, in PS1 pigs we observed an increase in expression of two NF-κB subunits p65 (RELA; 2 fold over 0 dpi, q<0.001) and p50 (NFKB1, 1.3 fold over 0 dpi, q<0.06), and the NF-κB regulon was shown to be over-represented in the SNEA analysis (Figure 6). As well, the gene for the NF-κB inhibitory subunit IκB (NFKB1A), which is a well-known target of the TLR signaling network through NF-κB, was up-regulated in PS1 pigs only (2.3 fold over 0 dpi, q<0.003).

Most importantly, the serum protein level of IFN-γ, the top ranked regulator for the up-regulated genes in PS1 animals, increased after ST inoculation and was positively correlated with *Salmonella* shedding levels [31]. IFN-γ produced predominantly by T lymphocytes and natural killer cells [80], is believed to prime macrophages to respond more vigorously to LPS. IFN-γ signaling also induces expression of several TLR signaling components, including TLR4 and MD2, both of which are up-regulated in the PS1 animals in response to *Salmonella* inoculation (TLR4: 5 fold over 0 dpi, q<0.02; MD2: 1.4 fold, q<0.04). IFN-γ has been proposed to remodel initial NF-κB signaling through both feed forward and feedback mechanisms [80]. First, IFN-γ amplifies TLR signaling by stimulating the expression of IRF1 and its downstream targets, which overlap with TLR signaling as IRF1 is also a target of NF-κB [41], [81]. SNEA analysis shows that the IRF1 regulon is significantly over-represented in PS1 pigs. Also, IRF1 mRNA is significantly up-regulated as well in these animals due to *Salmonella* inoculation (2.3 fold, q<0.004). Second, IFN-γ further activates proinflammatory pathways by suppressing IL10 expression, which stimulates STAT3-dependent suppression of TNF [80]. In PS1 pigs with high levels of circulating IFN-γ, IL10 RNA levels are nonresponsive to *Salmonella* inoculation, while STAT3 and TNF are slightly but significantly up-regulated in PS1 animals (1.49 fold over 0 dpi, q<0.01 and 1.65 fold over 0 dpi, q<0.08, respectively). Another set of STAT3 targets are SOCS genes, and SOCS3 is highly stimulated in both PS1 (6.4 fold) and LS1 (3.3 fold) pigs subsequent to *Salmonella* inoculation. However, the induction of SOCS3 mRNA is significant for PS1 pigs only (q<0.01), likely representing a negative feedback mechanism for IFN-γ signaling in PS1 animals.

Expression of TLR9, another gene positively regulated by IFN-γ through the actions of SPI1 and IRF8 [82], is slightly but significantly down-regulated in PS1 animals (1.53 fold compared to 0 dpi, q<0.04) and unchanged in LS1 animals. Porcine PBMC express TLR9 and respond to bacterial CpG DNA [83], with this TLR9 signaling inducing IFNG RNA expression in culture [84]. Human cells transfected with swine TLR9 and treated with CpG DNA increased cytokine-specific mRNA expression, including IFNG [85]. Mice infected with ST, as well as murine macrophages treated with LPS, also demonstrate increased TLR9 expression [86]. These observations are in contrast to the TLR9 response seen in PS1 pigs. Interestingly, inhibition of TLR9 signaling using an inhibitory oligonucleotide increased *Salmonella* replication in cultured macrophages [87], which is consistent with the lower levels of TLR9 expression seen in the PS1 animals. In our data, levels of TLR4 RNA were also increased in PS1 animals, suggesting that regulation of different TLRs is distinct in whole blood cells in vivo.

Combining our SNEA analysis results with the circulating cytokine data, we hypothesize that much of the difference in gene expression between PS and LS animals at 2 days post inoculation could be directly due to the higher levels of IFN-γ in the bloodstream. Elevated blood levels of IFN-γ would induce expression of IRF1 and other downstream components of the TLR and IFN-γ regulons. It would be interesting to test this hypothesis through measuring the correlation of IFN-γ signaling levels and gene expression response separately from *Salmonella* infection.

Overall, these results show that measurement of the transcriptomic response in a limited number of individuals with extreme disease outcomes identified substantially different responses of the major canonical immune pathways between these two extremes. As our gene expression data were quite extensive in the number of responsive genes, this experiment also provided significant new information for many poorly annotated transcripts in the porcine genome, as a correlation of their expression pattern to the known regulatory pathways described above may be a clue as to their regulation and function in the porcine immune response.

We also compared our response patterns with the data of Wurfel and colleagues [88]. This group quantified human whole blood responses for eight cytokine proteins from 102 patients to identify extremes in individual cytokine secretion after 6 hour LPS stimulation [88]. They then measured differences in global mRNA expression among three low and four high responders, cataloging genes that responded to LPS stimulation, as well as differentially expressed genes between the high and low responder classes. Comparing these results and our data, we found no overlap between genes differentially expressed between PS1 and LS1 animals at 2 dpi and genes differentially expressed between low and high cytokine secretors reported by Wurfel et al. [88]. The lack of overlap may be due to differences between *in vivo* responses to live bacteria and responses to a short-term LPS treatment *in vitro*. A smaller number of differentially expressed genes was observed between high and low responders by Wurfel and colleagues, as compared to the LS1 and PS1 animals, and this could also potentially explain the lack of overlap.

### Integrative analysis of miR-155 regulation with two important transcription factors in the porcine blood transcriptome response to *Salmonella*

**Figure 8:**
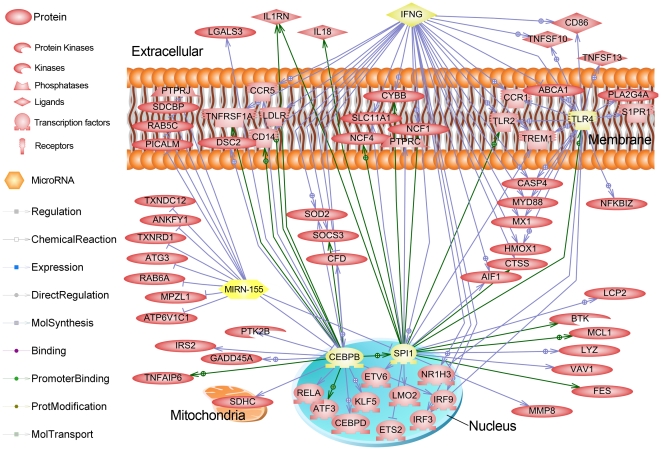
Common gene interactions of the IFNG, SPI1, TLR4, CEBPB and mir-155 (highlighted with yellow) gene expression regulation networks for up-regulated genes in the PS animals. In this figure, all entity relationships (protein-protein binding, promoter binding by transcription factor, known regulatory relationship, and other relationships) are shown for all up-regulated genes in the PS animals that were shown to be regulated by one of these five genes in the Sub-Network Enrichment Analysis. Green lines indicate promoter binding evidence; blue lines indicate regulation evidence only; all links reported by PubMed literature were obtained using Pathway Studio® (Ariadne). Note the large number of genes in common among the IFNG, SPI1 and CEBPB regulons, as well as that miR-155 negatively regulates both SPI1 and CEBPB.

Beyond the IFN-γ regulon, we further explored selected regulators showing large numbers of target genes in the differentially expressed gene lists, including TLR4, CEBPB, and SPI1, all of which we confirmed by q-PCR as up-regulated genes. The CEBPB gene product controls the expression of IL6, IL8 and other acute phase genes [89]. The CEBPB regulatory factor was among those genes up-regulated in porcine spleen upon *Haemophilus parasuis* infection [47]. The SPI1 gene product (PU.1) is well-known as a transcription factor controlling hematopoesis and stimulation of gene expression during the immune response [90], [91], including the TLR4 gene. Recently, the SPI1 gene was shown to be synergistically activated by NF-κB and CEBPB [92]. The SPI1 gene was found to be up-regulated at 2 dpi in both the LS1 and PS1 animals, while CEBPB is up-regulated only in the PS1 animals (2.2 fold, q<0.001). To visualize the commonly-regulated genes among these regulators, we collected the expression targets of IFN-γ SPI1, CEBPB and TLR4 and created a sub-network of all common genes in Pathway Studio®; the result is shown in Figure 8.

In addition, one of the miRNAs with significantly enriched targets within the up-regulated genes in PS1 animals is mir-155, which was down-regulated over 5-fold following ST challenge in persistent shedder pigs in a separate challenge population (PS3) (p<0.01). Recently, mir-155 has been reported to target (inhibit) the other two significantly enriched gene expression regulators, SPI1 and CEBPB [93], [94], [95], thereby explaining the expression pattern for much of the sub-network shown in Figure 8. The RNA expression level changes, confirmed by qPCR for TLR4, CEBPB, SPI1 and miR-155, provide substantial evidence to support the SNEA-generated hypothesis of the coordinated involvement of these factors in the regulation of the global gene expression responses introduced by ST inoculation. Our results add evidence of a direct connection between miR-155 and IFN-γ signaling [96]. Banerjee and colleagues showed that during CD4^+^ T cell activation miR-155 levels increase and promote Th1 differentiation, while suppression of miR-155 levels promoted Th2 differentiation. They demonstrated that lower levels of miR-155 increased IFNGR1 RNA levels and that the IFNGR1 mRNA had a functional miR-155 target sequence. Consistent with these regulatory interactions, we note that in PS1 animals (high IFN-γ low miR-155), IFNGR1 RNA was significantly increased after *Salmonella* inoculation (3.6 fold, q<0.002) while in LS1 animals the increase is less dramatic (1.8 fold, q<0.13). Alternatively, we could find no common targets of the cytosolic DNA-sensing pathway with the targets of the above regulators, thus this pathway is apparently relatively independent of those shown in Figure 8.

Finally, we believe this analysis shows that whole blood, in addition to being a practical and useful source of biomarkers associated with disease outcomes as has been shown for tuberculosis severity [51], may be an appropriate model to understand the regulatory mechanisms associated with the response to *Salmonella in vivo*, even though the RNA is derived from a mixture of cell types. We provided evidence for both transcriptional and post-transcriptional regulatory pathway involvement in the porcine whole blood RNA data collected herein. The present expression profiling data can also be a useful starting point for the identification of genetic variants that associate with the shedding phenotypes and that reside within candidate genes such as those differentially expressed between *Salmonella* shedding phenotype classes. Thus these novel data will be an excellent foundation upon which to build both of these approaches in the porcine species, an emerging model for the mammalian immune response.

## Supporting Information

Table S1 The *Salmonella enterica* serovar Typhimurium shedding counts and AULC (Area Under Log Curve) values for the 40 challenged pigs.
(XLSX)

Table S2 The statistical analysis results of the microarray data using a linear mixed model including fixed effects for shedding status, time, and interaction between shedding status and time.
(XLSX)

Table S3 Differentially expressed genes detected by the microarray (q value<0.05 and fold change>1.5 or <0.667).
(XLSX)

Table S4 Correlation analysis between the expression levels of transcripts with cell type numbers measured by Complete Blood Count Differential.
(XLSX)

Table S5 The qPCR results for the 21 genes in the 20 animals that were extreme for AULC values (LS1PS1 and LS2PS2).
(XLSX)

Table S6 The DAVID analysis results for the differentially expressed gene list.
(XLSX)

Table S7 The over-represented Innate DB pathways identified in the differentially expressed gene list.
(XLSX)

Table S8 The gene expression regulators whose targets were over-represented in the list of differentially expressed genes.
(XLSX)
